# Prioritizing Protecting Oneself Over the COVID-19 Virus Versus Other Health and Social Needs Among Older Adults Living Alone: A Qualitative Study

**DOI:** 10.1093/geront/gnae089

**Published:** 2024-07-24

**Authors:** Élise Develay, Lise Dassieu, Olivier Beauchet, Kevin Galery, Amélie Quesnel-Vallée, Sathya Karunananthan, Claire Godard-Sebillotte, Patrick Archambault, Cyrille Launay, Éric Tchouaket, Svetlana Puzhko, Paul Holyoke, Nadia Sourial

**Affiliations:** University of Montreal Hospital Research Centre, Montreal, Quebec, Canada; University of Montreal Hospital Research Centre, Montreal, Quebec, Canada; Department of Medicine, University of Montreal, Montreal, Quebec, Canada; Centre de Recherche de l’Institut Universitaire de Gériatrie de Montréal (CRIUGM), Montreal, Quebec, Canada; Centre de Recherche de l’Institut Universitaire de Gériatrie de Montréal (CRIUGM), Montreal, Quebec, Canada; Department of Equity, Ethics and Policy, McGill University, Montreal, Quebec, Canada; Department of Sociology, McGill University, Montreal, Quebec, Canada; Interdisciplinary School of Health Sciences, University of Ottawa, Ottawa, Ontario, Canada; Department of Medicine, Division of Geriatrics, McGill University, Montreal, Quebec, Canada; Department of Anesthesiology and Critical Care, Division of Critical Care Medicine, Université Laval, Laval, Quebec, Canada; Department of Family and Emergency Medicine, Division of Critical Care Medicine, Université Laval, Laval, Quebec, Canada; Department of Medicine, Division of Geriatric Medicine, Jewish General Hospital, McGill University, Montreal, Quebec, Canada; Department of Nursing, Université du Québec en Outaouais, Gatineau, Quebec, Canada; Department of General Practice and Family Medicine, University of Bielefeld, Bielefeld, North Rhine-Westphalia, Germany; Department of Health Science, University of Toronto, Toronto, Ontario, Canada; University of Montreal Hospital Research Centre, Montreal, Quebec, Canada; Department of Health Management Evaluation and Policy, School of Public Health, University of Montreal, Montreal, Quebec, Canada

**Keywords:** Distancing measures, Pandemic, Priorities, Protection

## Abstract

**Background and Objectives:**

People aged 65 and older, deemed most “vulnerable” by public health, were targeted by the coronavirus disease 2019 protection measures, which sought to minimize physical contact and social activities. Older adults living alone were particularly affected by these measures. However, such measures meant to protect the older population may not have necessarily reflected older adults’ individual prioritization choices. This study therefore aimed to understand how protecting oneself over the virus was considered in the prioritization of other health and social needs of older adults living alone during the pandemic.

**Research Design and Methods:**

This study adopted a qualitative design. A total of 17 semistructured interviews were conducted between May 2021 and June 2022 with older adults living alone. All interviews were audio-recorded and transcribed verbatim. A reflexive thematic analysis as defined by Braun and Clarke was performed.

**Results:**

Our analysis showed 2 forms of prioritization across 2 themes. This first theme focused on participants who reported prioritizing protecting themselves over the virus by limiting in-person contact and activities. The second theme showed that although several participants reported that protecting themselves over the virus was important to them, the prioritization of this need was not shared by all and, in some cases, evolved over the course of the pandemic.

**Discussion and Implications:**

Our study demonstrated heterogeneity in the prioritization of older adults needs. Future public health recommendations should consider these variations in the needs and priorities of older adults when determining public health measures.

## Background

Aging is associated with an increase in chronic illness, disability, and changes in social activities (Chapleski et al., 1997; Desrosiers et al., 2009; Kergoat & Légaré, 2007). Consequently, the needs of this segment of the population may be more numerous and specific over time.

In this study, we refer to needs as “the measurable discrepancy existing between a present state of affairs and a desired state of affairs as asserted either by an ‘owner’ of need or an ‘authority’ on need” (Beatty, 1981, p. 40). Many of the health needs of older adults identified in the literature have been based on physical needs, such as mobility, function, eyesight, hearing, and mental health needs such as psychosocial care and support (Abbott & Sapsford, 2005; Cheraghi et al., 2021; Kossioni, 2023; McCausland et al., 2021; Van Aerschot et al., 2022). Social needs in older adults identified in the literature have included social activity needs, companionship, intimate relationships, personal relationship with carer, and daytime activities (Cheraghi et al., 2021; McCausland et al., 2021; Van Aerschot et al., 2022). A systematic review identified “diversity, proximity, meaning of the relationship, and reciprocity” as key characteristics when analyzing the social needs of the older adults (Bruggencate et al., 2018).

While there is a wealth of literature on the needs of the older population, evidence remains scarce on how they prioritize their needs at an individual level. Meyerhof et al. (2022) found that many older adults generally prioritize their health over other life domains such as “partnership, friendship, standard of living, occupation, and family.” In particular, they found that women and persons with low health status prioritized health more than men and those with higher health status. A study by Strout et al. (2018) also supports that older adults prioritize their health over other needs such as their social needs. The authors found that the top three priorities reported by older adults were physical health followed by social and emotional well-being (Strout et al., 2018). Of interest, in a study by Junius-Walker et al. (2019), when asked about how they prioritize their health needs, older adults explained that health needs that allowed them to continue their social activities were especially important.

The coronavirus disease 2019 (COVID-19) pandemic may have required older adults to reconsider how to prioritize their health and social needs in the face of the COVID-19 virus. Several jurisdictions implemented measures to protect the population from the virus, including the closure of socializing spaces, stay-at-home measures, and curfews (Mathieu, 2020). People aged 70 and older, deemed most “vulnerable” by public health authorities, were particularly targeted by the prevention measures in terms of their intensity and duration, which sought to minimize physical contacts and social activities (Centers for Disease Control and Prevention, 2023; Ministère de la Santé et des Services Sociaux, 2020). Older adults living alone were particularly affected by these measures (Savage et al., 2021; Wong et al., 2020).

Government measures meant to protect the older population may not have necessarily reflected older adults’ individual prioritization choices. There is a paucity of literature exploring prioritization of needs in older adults during the pandemic. Mello et al. (2022) identified priorities of older adults during the COVID-19 pandemic based on data collected from outpatients and hospitalized patients. The highest priority reported by respondents was to “protect oneself over COVID-19 and survive the pandemic” (29%). However, these results did not provide in-depth understanding of how older adults prioritized their needs in this context. In addition, there is a lack of evidence on prioritization of needs for older adults living alone during the pandemic who have been shown to be more likely to report loneliness during social distancing induced by government measures (Emerson, 2020) and for whom the need for social relationships, for example, may have influenced prioritization choices.

Understanding how older adults, in particular those living alone, prioritized protection over the virus from other health and social needs during the pandemic is critical to ensuring that future government measures and the planning of service delivery consider the preferences of older adults living alone, which may be specific in a pandemic context.

This study therefore aimed to understand how protecting oneself over the virus was considered in the prioritization of other health and social needs of older adults living alone during the pandemic.

## Method

### Study Design

This study adopted a qualitative case study design (Creswell, 2013) as it allows for an in-depth understanding and the acquisition of rich knowledge by integrating context as an influence on data. This study involved 17 semistructured interviews conducted between May 2021 and June 2022 with older adults in the greater Montreal and Quebec City areas of the province of Quebec, Canada. This study was derived from a larger study that explored social isolation and loneliness in older adults living alone during the pandemic (Sourial et al., 2023).

### Participants and Recruitment

Participants for this study were recruited among older adults who completed ESOGER (French acronym for SOcio-GERiatric Evaluation), a telephone-based online risk assessment tool for adults aged 65 and older, covering their physical, social, mental, and cognitive vulnerabilities (Beauchet et al., 2020; Sourial et al., 2023). ESOGER was distributed during the COVID-19 pandemic to various health and community entities across the broader Montreal and Quebec City regions, including geriatric clinics, social services agencies, and community healthcare centers. Its purpose was to assist healthcare practitioners in identifying the needs of older adults with vulnerabilities. The tool has been previously validated and later modified for utilization amidst the COVID-19 crisis (Beauchet et al., 2020). For this study a purposive sampling method was used to recruit participants covering a range of demographic characteristics including age, sex, and rurality to ensure a diversity of experiences. Eligible participants were community-dwelling individuals aged 65 years or older, living alone and able to speak French or English. We chose to recruit over a broad period of time (13 months) in order to capture a diversity of prioritization choices at different moments in the pandemic. Individuals who met the eligibility criteria for the current study were contacted to participate in our study. Participants were offered a $25 compensation for their participation.

### Procedure

Individual semistructured interviews were conducted by phone by E. Develay and partly by N. Sourial. Interviews were audio-recorded with participants’ consent. Given this study stems from a larger project exploring social isolation and loneliness in older adults living alone during the pandemic, the interview guide included open-ended questions on various topics related to the pandemic such as social relations, health experiences, and perceived needs (see Supplementary Material - Text 1). Open-ended sociodemographic questions were asked at the end of interviews. Interviews lasted between 25 and 113 min (average = 46 min and median = 42 min). Interviews were conducted in French and English and the selected quotes were translated into English by one of the bilingual coauthors. All interviews were transcribed verbatim by a professional with the deidentification of all names, dates, and places.

A total of 17 individuals participated in the study. Two team members (E. Develay, N. Sourial) made the decision to stop recruiting based on information power, meaning when sufficient quality and diversity of data were collected (Malterud et al., 2016), as well as when a certain extent of redundancy was reached (data saturation).

### Data Analysis

Three team members conducted the data analysis (E. Develay, N. Sourial, and L. Dassieu). We used an inductive reflexive thematic analysis following the six phases of thematic analysis as described by Braun and Clarke (Braun et al., 2019; Clarke & Braun, 2018). First, we began by familiarizing ourselves with the data (1), that is, we read all the data and memos written after each interview to get an overall picture of the raw data. We then generated initial codes (2). Searching for themes (3) began once we coded the entire data set. We then reviewed and refined candidate themes (4) meaning we ensured the coherence of pattern of data for each theme and ensured that there was no overlap. Once we had a general plan of our themes and subthemes, we started to write and define them (5) to arrive at the current study results (6). Several meetings with the research team during the analysis process allowed us to validate the codebook and find our final themes and subthemes based on the data. Diaries were written throughout the analysis process to enhance the researchers’ reflexivity in interpreting the data. Dedoose software (Dedoose, 2016) was used for data analysis.

#### Process of coding and generating themes

Initially, we coded with the objective of exploring the social and health needs of older adults during the pandemic. First, we coded with a very open initial coding, to synthesize the data and have easy access to the information. For example, we began coding with first-level codes for “Social Needs,” “Health Needs,” and “Need of protection over the virus.” We used subcodes to detail each first-level code, such as “Feeling socially included” to specify the “Social Needs” code. When we were unsure about the relevance of information, we discussed it with the team as the coding progressed. For example, the subcode “Not feeling vulnerable” seemed to be close to our topic, but we could not initially place it in relation to our codes at this stage of the coding. When in doubt about the relevance of subcodes, it was decided to keep them and reassess their relevance later in the analysis process.

If we did not agree on how to interpret the data, we determined whether the reasons were related to differing perspectives linked to the authors’ fields of expertise (e.g., sociology vs public health). In such cases, we then tried to integrate the different perspectives in order to nuance the interpretations. The codes on prioritization were assigned at a more interpretive level, when there was a dimension of putting one need before another in the participants’ narrative. We used, for example, codes like “Prioritization of health needs.”

Once we obtained codes on prioritization that allowed us to both identify different types of prioritization and to understand the links between them, we decided on themes for the different types of prioritization. The links we found between the codes allowed us to identify our subthemes. For example, we were able to combine the codes “missed prioritization” and “not feeling vulnerable” to create the subtheme “Denied desire to prioritize in-person social contacts” (see Supplementary Material - Figure 1).

### Characteristics of Participants

Most participants were female (12/17), and reported living in an urban area (12/17) and received no governmental income supplement (11/17). Participants’ sociodemographic and professional characteristics are shown in Table 1.

**Table 1.** Participant’s Sociodemographic Characteristics

Characteristics	Participant (*n* = 17) *n* [%]
Urban	12 [71]
Age	
65–74 years old	4 [23.5]
75–84 years old	4 [23.5]
85–94 years old	8 [47.1]
≥95 years old	1 [5.9]
Gender	
Women	12 [71]
Men	5 [29]
Other	0 [0]
Canadian born	10 [59]
Education level	
Secondary school	7 [41]
University	10 [59]
Income	
Government income supplement	6 [35]
No supplement	11 [65]

### Ethics

This study was approved by the Research Ethics Committee of the CHUM (20.123) and followed the Standards for reporting qualitative research (O’Brien et al., 2014).

## Results

We uncovered two broad themes based on the reported experiences of participants: (1) the prioritization of the need to protect oneself over the virus over other health and social needs and (2) the prioritization of social needs over the need to protect oneself over the virus.

### Theme 1: Prioritizing the Need to Protect Against the Virus Over Other Health and Social Needs

This first theme focused on participants who reported prioritizing protecting themselves over the virus by limiting in-person contact and activities. The need of protection over the virus was mainly characterized by our participants as a worry about catching the virus. The first subtheme showed how the prioritization choice came about and why, while the second subtheme showed the consequences of such prioritization.

#### Choosing to protect against the virus over the need for in-person contacts

During the pandemic, some of the participants indicated that they had to choose between two contradictory needs: the need for in-person contact with their loved ones versus the need to protect themselves over the virus:

Interviewer: And during the pandemic, were you able to receive visits from outsiders, even if you didn’t go out?

Participant: No, I refuse visits. My nephews and nieces in Montreal, they bring me food, they always leave it outside the door. (Jin, 86)

Although they recognized that their need for in-person contact was strong and admitted they missed it, they prioritized protecting themselves against the virus:

The most difficult thing is not being able to see my children and grandchildren, my friends, but I imposed this on myself and I simply respected my choices. (Marie, 66)

This need to protect oneself over the virus sometimes went beyond the government measures in place. Several participants continued to restrict their outings to avoid contracting the virus even when health measures allowed public places such as cinemas, theaters, and concert halls to reopen.

Even then I think I won’t go [to the movies]. Yes, I don’t feel comfortable being in a closed theater. What I understood this week is that they will reopen, there will no longer be any restriction on the number of people in a place, in a room. Not all people respect the safety measures because there are people who will be unpleasant, who will not wear their mask. No, I don’t like that. (Marie, 66).

When asked about the reason for restricting their outings, participants explained that they considered themselves vulnerable because of the risk that COVID-19 could pose to their health.

It was a little worrying—this COVID thing—because I already have fibrous lungs and I suffer from asthma, so it’s certain that I was rather vulnerable. (Francoise, 92)

Although participants in our study lived alone, in most cases, limiting in-person contact did not seem to induce feelings of loneliness. According to them, this could be explained by the fact that they maintained their long-distance contacts (e.g., telephone calls, video calls), had already become accustomed to living alone before the pandemic, and had already developed routines that ensured that social isolation did not lead to feelings of loneliness.

Still, living alone for 12 years, I have my “routine”. I am used to living alone for 12 years since my spouse passed away, it is part of my daily life to be alone. With the children at work, the social life was very cut off. It was hard not to see my friends too, because we often went out for dinner or something like that, to the theater, to the movies, to entertain each other. But now we had cancelled all that. But we talked daily on the phone. We have the habits of women my age, I guess. (Sylvie, 75)

#### Choosing to protect over the virus at the expense of certain health needs

In some cases, participants chose to prioritize the need to protect themselves over the virus at the expense of managing some of their chronic conditions. This prioritization in turn had an impact on their mobility and mental health.

Although participants explained that they continued to attend their medical appointments, their physical conditions (e.g., osteoarthritis) requiring self-management such as physical activity (e.g., walking) were not always prioritized. The protection over the virus therefore came at the expense of managing these physical conditions.

I refuse visits from all my nephews and nieces in Montreal. I can’t go out because of my health, and I’m becoming more and more … before with the help of a cane I could go out but now I can’t … (Jin, 86)

In addition to the physical health consequences, this type of prioritization had a consequence on mental health for some participants. Some noted that they had lost the motivation to leave their homes as a result of staying at home, even though government measures allowed it.

Now that there’s a little more freedom, more tolerance to do something, I feel like I’ve caught a little bit of … how can I explain it, depression. Yes, because I feel that I don’t want to do things, I don’t want to go out. Even if we can go out, I’d like to stay at home. (Theresa, 71)

### Theme 2: Prioritizing the Need for In-Person Social Contacts Over Protection Over the Virus

Even if several participants said that protecting themselves over the virus was important to them, the prioritization of this need was not adopted by all and, in some cases, evolved over the course of the pandemic. The first subtheme showed how the prioritization choice came about and why, while the second subtheme showed why some participants were denied to make such prioritization and its impact on them.

#### Choosing in-person social contacts over protection from the virus

This prioritization choice was made with a thoughtful balancing between their needs. For example, the participant below explained how she wrote off her apartment as a potential meeting place but still met outside to meet her social needs:

Interviewer: Why do you prefer to see your children in person rather than call them, for example?

Participant: It’s because when I don’t see them, I miss them. They didn’t come either so they wouldn’t contaminate me and I didn’t want them to come either. Downstairs in my apartment, outside, I didn’t mind but I didn’t want people in my apartment. And I had canceled the housekeeper, because I didn’t want to catch this. I was in a bad position. (Jeanne, 89)

Some participants prioritized their need for in-person contact at the onset of the pandemic, sometimes by ignoring government measures in effect.

We may have cheated the system a little bit … when (my husband’s niece and nephew) wanted to come see me, they would come. And I would arrange at that time for them to go through the front door. (Josseline, 83)

In this case, this priority was explained as a social need stronger than the fear of being infected.

I was having my niece over. The need was there and it was stronger than the fear. (Josseline, 83)

The majority of participants kept in touch with their loved ones during the pandemic, either by calling each other on the phone or using video calls, but while these media allowed for the ability to connect at a distance, they did not replace the closeness of in-person social contacts, according to the participants.

Interviewer: What was the difference between calling her on the phone and going to see her?

Participant: I don’t know. There is more closeness when we are in front of each other I mean … because we confide in each other a lot. (Marc-André, 71)

Moreover, participants reported that in-person social contact brought a certain “human touch” allowing, for example, to have a physical contact with a loved one (e.g., a hug, an embrace).

So uh, obviously we talk on the phone, but that doesn’t solve the fact that we are far away, we need this human warmth. Which we don’t have anymore. Human warmth is being able to give each other a hug, to be able to hold someone’s hand, you know? So it’s that you feel like part of a family, if you like, but there you lose your reference points at a certain point … you say to yourself, yes, we’re close, but how close are we? (Josseline, 83)

In this case, the fact that remote relationships could not support the needs associated with in-person relationships was a reason for the prioritization of social needs.

The lack of physical contact was also reported as a reason for shifting priorities during the pandemic to prioritize the need for in-person relations.

We were paying attention, each on our own, and then at a certain point when we see the little baby who is starting to walk and who comes towards you so proudly, to show that “I can walk” I fell to the ground and I asked “can I take him?” I took him in my arms and I cried. And then I said “that’s enough”. When I looked at the parents, I said, “That’s enough, come and hold me too. It was a beautiful moment, a very beautiful moment". (Marie, 66)

Another reason reported by participants explaining this shift of priority was a decrease of perceived vulnerability to the virus as the pandemic evolved allowing them to prioritize their need for in-person social contact.

At that time [at the beginning of the pandemic], it was bad because the pandemic was starting and the cases were increasing every day. There was more fear. Now I think it’s more relaxed because the number of cases is going down and I think we’re coming down a little bit *…* because I’ve been tested three times and every time it’s been negative, I feel like I’m a little bit more resistant to having it. (Theresa, 71)

The prioritization of the need for protection over the virus over social needs was therefore not consistent throughout the pandemic. Of note, changes in priorities were only reported as a shift from prioritizing the need for protection over the virus to the need for in-person social contact, and not the other way around.

Now it’s okay, now I’m quieter and more relaxed but at first I would go home and run, run, run to get what I needed to get out. It means that I spent some, as I can say, very upset and stressed because of that but I think the most important thing is the social contact. The social contacts I can’t visit, I can’t go to the gym, I can’t entertain at home. I feel really lonely sometimes. (Theresa, 71)

#### Denied desire to prioritize in-person social contacts

Finally, several participants reported wishing to prioritize their social needs but being unable to do so because their loved ones decided not to visit them so as not to risk infecting them. Participants reported not feeling especially vulnerable to the virus and willing to accept associated risks.

Interviewer: Do you feel vulnerable to COVID-19?

Participant: Not really. If I got it, that’s the way life would be. (Philippe, 88)

These participants reported experiencing a disconnect between the way they saw themselves and the way they were seen as vulnerable by the government or their loved ones, resulting in unwanted protection against the virus.

[My daughters] had taken the position of not coming to see me so they wouldn’t infect me. (Rose, 87)

Even when measures were loosened, allowing for small group gatherings while maintaining physical distancing, some participants reported facing difficulties because of this distancing, which went against their willingness to prioritize in-person social contact. This unwanted distancing resulted in a sense of differential treatment from the rest of the group, leading in some cases to a feeling of being left out. So here the prioritization of the need for in-person social relations could not be achieved on the participants’ terms.

We are allowed to stay together, separated on the patio, but for me it was very strong, like a shock, I had to eat at one side of the table and they were eating at the other side of the table. All the things that are for me are plates especially with my portion, I can’t touch the other portion of the salad for the rest of the family. That’s one thing that really, really got to me. My daughter says to me, “Mom, this is a precaution for you because you are older than them”. (Theresa, 71)

These in-person contacts were not enough so long as there was still physical distancing: in some cases, the physical distancing was so distressing that the participant preferred to withdraw from in-person gatherings.

That’s why, for example, now my son says to me: “You can come on the weekends. Now you can come from [province 1 of Canada] to [province 2 of Canada] and [province 2 of Canada] we can go to [province 1 of Canada], come on the weekends now that we can have I think 10 people in the house”. No, I’m not going. (Theresa, 71)

## Discussion and Implications

Our study aimed to understand how protection against the COVID-19 virus was prioritized over other health and social needs of older adults living alone during the pandemic. Our results showed that participants prioritized in two ways: the prioritization of the need to protect over the virus over both health and social needs and the prioritization of social needs over protection over the virus. Our findings stand out in showing that, despite the pandemic context, protecting over the virus was not always prioritized by older adults living alone, with some prioritizing social contacts at the risk of contracting the virus.

The prioritization of the need to protect over the virus espoused by participants sometimes extended beyond government measures, in cases where participants perceived themselves to be particularly vulnerable in relation to the risks the virus could pose to their health. This finding was consistent with Bearth et al. (2021) who reported that older adults’ acceptance and adherence to COVID-19 protection measures were particularly high for people with preexisting condition. Surprisingly, despite living alone, the majority of participants did not report feeling lonelier because of the pandemic since they were already living alone before pandemic. Interestingly, a study by Bundy et al. (2021) showed that when pandemic-related loneliness was seen as an acceptable consequence of social distancing to protect against the virus by older adults, they were more willing to limit their social ties and, this, even when they reported feeling a lack of intimate social relationships.

Nevertheless, our second prioritization theme showed that under certain circumstances, the social need for in-person contact in older adults living alone can take precedence over other needs. This was true for those who no longer felt vulnerable to the virus, and also when the need for in-person contacts was stronger than the risk of catching the virus (e.g., lack of “human touch” and closeness of in-person social contacts). This is in contradiction with the findings of Strout et al. (2018) and Meyerhof et al. (2022) who examined prioritization in general older adults prior to the pandemic as well as Mello et al. (2022) who surveyed both older inpatients and outpatients with multiple comorbidities, including dementia, during the pandemic. These authors found that, in both contexts, health was prioritized over other needs. Specifically, Mello et al. (2022) found “protecting oneself over COVID-19 and surviving the pandemic” to be the first priority among the respondents. Our findings therefore demonstrate a heterogeneity in the prioritization of older adults needs, which may be particularly different among older adults living alone. Indeed, several studies have pointed to the importance of a proximity network for older persons living alone who are often more likely to suffer from social isolation and loneliness (Cudjoe & Kotwal, 2020; Emerson, 2020; N. Sourial et al., 2023). As recommended by Lewis and colleagues (2022), this emphasizes the importance of strengthening infrastructures that allow the older persons living alone to socialize (convenience stores, community centers, etc.) during potential future pandemics.

Moreover, this second prioritization theme showed that although government measures were aimed at protecting the health of older adults, they were not always aligned with participants’ prioritization of the need for in-person social contact. Many of our participants felt labeled “vulnerable” by either government measures or loved ones in a way that was inconsistent with their self-perception, leading in some cases to feelings of social exclusion. Falvo et al.’s (2021) article on the experiences of older adults during the onset of the pandemic in Switzerland had anticipated that this situation could arise from considering all older adults homogeneously at risk, which was deemed a reductive and therefore stigmatizing approach. Similarly, studies reported that COVID-19 plausibly exacerbated ageist attitudes (Barth et al., 2021; Fraser et al., 2020).

Several of our participants disclosed that they had breached distancing measures in order to receive their loved ones at home. This is in contrast to the literature reporting that, even though older adults found distancing measures difficult to live with, notably because of isolation and lack of physical contact (Fiocco et al., 2021), they did not report having violated government measures (Falvo et al., 2021; Fiocco et al., 2021; Greenwood-Hickman et al., 2021; Portacolone et al., 2021). Our divergent result could be explained by the fact that our study was conducted after the first year of the pandemic, which potentially brought a certain hindsight (e.g., with the arrival of the vaccines), thus alleviating general fears associated with the virus, and allowing more confidence to speak freely about nonconforming behavior.

The diversity of priorities observed among older adults in our study highlights that health is not always at the top of their priorities, with some choosing to put their social needs first. Moreover, participants’ dissatisfaction with “vulnerability” labels imposed by public health measures and loved ones speaks to the unintended consequences of pandemic measures on ageism and stigmatization toward the older adults. It is therefore important to consider that the prioritization of needs among older adults are heterogeneous (Dassieu & Sourial, 2021), and future public health recommendations should consider these variations in determining public health measures that respect the choices of older adults rather than basing risk solely on age. Sleap et al. (2018) suggested that older adults should be given more decision-making power when developing health measures by involving them in decisions about their own needs. Moreover, given the dynamic nature of prioritization, their involvement could be done repeatedly to capture changing prioritization.

Our findings revealed circumstances in which the prioritization of protection against the virus had an impact on physical and mental health. To respect the health needs of older adults who choose to protect themselves over the virus, additional resources may also be needed in the future to minimize the deterioration of their health status. One strategy could be to promote the use of telemedicine to prevent service disruptions and prioritize older adults’ access and literacy of this modality. For example, the use of online platforms has been proposed as a solution to maintain a level of physical activity during distancing measures (Garfin, 2020).

### Limitations

This study has some limitations. First, we did not collect information on participants’ health conditions. It would have been interesting to combine this information with our results on the prioritization of healthcare needs. Second, despite recruitment efforts, by restricting the sample to those who could speak English or French, we are not capturing the experiences of older adults who do not speak either of these languages and as a result may in fact face additional challenges in terms of isolation. Third, the study period occurring approximately 1 year after the start of the pandemic could have created difficulties associated with the recall of information or events in the earlier phases of the pandemic. Fourth, it is also important to mention that the individual experiences reported in this study are influenced by the political and social context in which individuals were immersed, as well as their social contacts. Thus, prioritizations should be understood as the product of a context, interactions, and individual choices (Bruch & Feinberg, 2017).

Even though we identified several reasons to explain the prioritization of our participants, our results could not provide an understanding of the mechanisms behind the prioritization process. Future research should be carried out to shed light on this broader theme. For example, it would be interesting to question whether beliefs and trust in the medical system played a role in prioritizing the need to protect against the virus.

## Conclusion

Our study demonstrated heterogeneity in the prioritization in the needs of older adults living alone. While some prioritized protection over the virus over their in-person social contacts or their need for physical activity, others prioritized in-person social contacts. Future public health recommendations should consider these variations in the needs and priorities of older adults when determining public health measures. Older adults, and perhaps especially those living alone, should be involved in this decision-making process, which could be repeated to capture changing needs, given the dynamic nature of prioritization.

## Supplementary Material

gnae089_suppl_Supplementary_Material

## Data Availability

In accordance with the requirements of our research ethics boards, study data cannot be readily shared. However, data transfer agreements can be put in place at the request of interested researchers for replication purposes.
