# Supplementary material for: Prioritizing Protecting Oneself Over the COVID-19 Virus Versus Other Health and Social Needs Among Older Adults Living Alone: A Qualitative Study
Source: Gerontologist. 2024 Jul 24;64(9):gnae089. doi: 10.1093/geront/gnae089 (PMC11342055; doi:10.1093/geront/gnae089)

**Supplementary Material**

**Supplementary** **Text 1 - Interview guide**

**Introduction - General situation**

- Can you tell me how the pandemic has impacted your social contacts?

- How have your social contacts changed during the pandemic?

**Experience of Isolation and Social Relationships during the Pandemic**

**Isolation 'objective' (social network)**

-Who are you currently living with?

-How often are you in contact with your loved ones?

-Are you satisfied with these contacts? What are your reasons for being satisfied?

- Have you or someone close to you had COVID-19?

-What was the impact on your social contacts?

-How do you think distancing measures and the pandemic impacted your well-being?

-What have been the changes in this (loneliness) feeling, during/compared to before the pandemic?

**Perceived isolation (feeling isolated)**

-On what occasions did you feel isolated during the pandemic?

-What did you usually do when you felt isolated?/ What makes you not feel alone?

-What impact did this isolation have on your daily life?

-What advice would you give to someone who is lonely?

-Did the vaccine change anything in your social contacts?

**Health experiences during the pandemic and interventions received**

- What are the impacts of the pandemic on your physical/mental health?

-Have you benefited from interventions to help you with your isolation? What were these interventions?

-What did you think of these interventions? How did they help you?

**Perceived Needs**

-What do you think you need most during the pandemic period to prevent against isolation?

-What health/social services would you like to have, relative to your isolation?

-What are your concerns about this pandemic?

-What are your hopes and expectations regarding the current pandemic situation?

**Conclusion**

-Do you have anything else to add that we haven't talked about? Are there any topics that you feel are important and that you would like to cover to conclude the interview?

**Supplementary Figure 1 – Example of process of coding and generating themes**


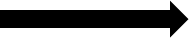

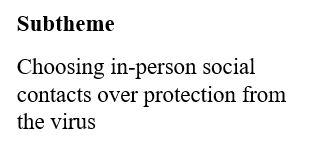

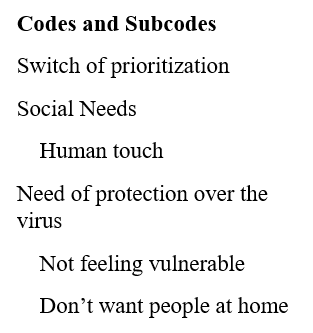

Supplement: gnae089_suppl_Supplementary_Material [file gnae089_suppl_supplementary_material.docx]
